# Asthmatic Mixture

**Published:** 1875-09

**Authors:** 


Asthmatic Mixture.—The following asthmatic mixture is much used in Roosevelt Hospital:

| | |
|---|---|
| Hoffman's Anodyne, . . . . | ℨj. |
| Tr. Belladonna, . . . . . | gtt.xv. |
| Potass. Iodid, . . . . . . | gr.v. M. |

S. One t.i.d.

It sometimes happens that the iodide gives rise to nausea; if so, it is dropped from the prescription.

CHRONIC BRIGHT'S DISEASE.

A favorite pill with a visiting physician, to be used in the general treatment of this affection, consists of

| | |
|---|---|
| ℞.—Hydrarg. Bichlorid., . . . . | gr.1-20. |
| Quinia Sulph., | |
| Digitalis Pulv., aa., . . . . | gr.i. |
| Belladonna Ext., . . . . | gr.$\frac{1}{4}$. |
| M. | |

S. One t.i.d.

### CHRONIC BRONCHITIS.

The administration of the following has been attended with a fair amount of relief in this class of cases:

℞.—Oleum Lini, . . . . . ℥ij.

Mucilagi Acaciæ, . . . . . ℥ijss.

Syr. Tolu, . . . . . . . ℥j.

Ol. Terebinth, . . . . . ʒiij.

Aquæ Cinnamomi, ad., . . . ℥viij.

M.

S. ℥ss. t.i.d.

### LINIMENT.

The following is quite a favorite for the relief of those cases with which every practitioner is disturbed, and need not be specified:

℞.—Tr. Aconit Rad., . . . . . ʒij.

Oleum Olivæ, . . . . . . ℥j.

Morphia Muriati, . . . . gr.vj.

Chloroformi, . . . . . . . ʒj.

M.

### CATARRHAL AFFECTIONS.

The following is a favorite prescription for catarrhal pneumonia, bronchitis, etc:

℞.—Ammon. muriat.,

Potass. Chlorat. aa., . . . . . ʒij. and ℈ij.

Aq. Aurant. Flor., . . . . . ʒij.

Syr. Simplicis, . . . . . . ℥ij.

Aquæ, ad., . . . . . . . ℥iv.

M.

S. Two teaspoonfuls t.i.d.

—*Med. Record.*

